# Case of Lithotomy

**Published:** 1873-01-01

**Authors:** H. Wardner

**Affiliations:** Cairo, Ill.


### A CASE OF LITHOTOMY.

BY H. WARDNER, M. D., OF CAIRO, ILL.

On the 31st of October last, in answer to a telegram, I visited a lad of nine years, at Walbridge, in Pulaski county, who was supposed to be suffering with a stricture. I found the patient much emaciated, frequently urinating, and suffering most excruciating agony at every attempt; his voice was hoarse from crying; he was feverish at times; had but little desire for food; and constantly calling for something to stop the pain. He was almost constantly pulling at the prepuce, which had become much elongated in consequence. As a result of his frequent violent straining and the pelvic tenesmus, the rectum was somewhat prolapsed, and the fœces were passed at nearly every effort to void the urine. His health had been gradually failing for over a year, but it was only four or five weeks since these more acute and violent symptoms had set in.

He was placed under chloroform, and a gum catheter introduced into the bladder without encountering any stricture. The finger was then introduced into the rectum and pressed against the neck of the bladder, so as to feel the catheter. A violent tenesmic contraction of the bladder followed, forcing the urine out suddenly, both through and *around* the catheter. At that moment I felt a body settle into the neck of the bladder, apparently about the size of an almond. Upon moving the catheter about a little, it disappeared and was not afterwards felt at that time.

I was not provided with a sound or metallic catheter of suitable size for the case, and consequently could not speak with an absolute certainty; but my diagnosis was stone in the bladder, most probably, Drs. J. W. Mott and — Purdy differing with me, in thinking that the almond-shaped body was the result of the tenesmic contraction, and that it disappeared as the spasm relaxed. My opinion was communicated to the parents; but I requested another opportunity to examine the patient before recommending any decisive action in the case.

On the 17th of November last, the father, Mr. A. Stevenson, came to my office and requested me to go prepared to remove the stone, which he had come to believe existed. I saw the patient the same evening, and had no difficulty in finding the stone with a metallic flexible sound. By moving the sound over the surface, I judged it to be at least an inch in diameter. The time for operating was set at eight o'clock next morning, *i. e.*, November 18th, and a messenger sent for Dr. J. W. Mott to assist.

At the appointed time the patient was anæsthetized by Dr. Purdy, who kept up the administration as required during the operation. The patient was placed in the usual position, on a common table, and tied. The stone was again readily detected with the grooved staff, upon its introduction into the bladder. Dr. Mott held the staff firmly up against the pubis, and I performed the lateral operation. After the first incision, which divided the more superficial tissues at once, I proceeded slowly, with the index finger of the left hand pressing the rectum aside and guarding the edge of the bistoury, using the knife only when the parts were not readily separated by the finger or the handle of the scalpel, until the membranous portion of the urethra was reached. The staff was then lowered a little, so as to bring it readily against the finger, and the point of the knife carried through the urethra into the groove. The probe-point of the lithotomy-knife was then passed through the slit and safely lodged in the lower termination of the groove. The staff was then drawn up against the pubic arch, seized, and with the knife carried into the bladder, as recommended and practiced by Professor Pancoast, when a gush of urine occurred. The staff and knife being withdrawn, the opening was easily dilated with the finger, although apparently not more than three or four lines in extent. The stone was readily found by the finger, lodged in a sort of pocket behind the pubis. It was dislodged, and fell upon the posterior wall of the bladder, when, upon attempting to seize it with forceps, it again slipped into the pocket. The hand of the assistant was then pressed firmly over the abdomen behind the pubis, when the stone was readily seized with a pair of small-sized forceps, and, by a little careful manipulation, removed. The hæmorrhage during the operation did not exceed an ounce. The wound and bladder were then syringed with warm water, and a female catheter introduced through the wound, and fastened by a waist-belt and tapes, and the patient placed in bed and given an anodyne.

The subsequent history of the case has been furnished me by Dr. Mott, who is entitled to much credit for his careful subsequent treatment of the patient.

Nov. 19th, 10 A. M.—Patient has rested well; slept about half the time since the operation without any repetition of the anodyne; pulse 106; respiration natural; urine discharging freely through the catheter; no hæmorrhage, tenderness, nor inflammation. I think he is doing remarkably well. Says he is bound to have that stone. No action of bowels; appetite poor.

Nov. 20th.—Condition much the same as yesterday; gave ten drops of laudanum twice a day, to keep bowels in check; syringed wound with carbolic acid wash, three grains to the ounce; removed catheter.

Nov. 21st.—Doing remarkably well; appetite returned; bowels still quiet; pulse 84.

Nov. 22d.—Rests well; pulse 90; urine discharging freely through the wound; some tenderness over bladder; bowels not moved; use of chloroform suspended; appetite good.

Nov. 23d, 11 A. M.—Patient suffering very much since six o'clock last night. I find him screaming with pain; pulse about 80; bowels full and hard; no urine passing. We gave him some chloroform, and introduced the female catheter through the wound into the bladder, when he passed considerable urine through wound and catheter, and had a large evacuation of the bowels. Five minutes later he had another very large fœcal discharge; then went to sleep, with much less distension of abdomen; pulse 84; no unnatural heat about him. At one o'clock P. M., he awoke, suffering intense pain. I suspect the possibility of another stone. "Come out to-night, if you can."

I visited the patient, as requested, and found him very comfortable, and the Doctor a little annoyed that he had called me out to the case, but I was glad he did so.

The rectum being so distended with feces, it pressed the walls of the wound at the neck of the bladder together, and closed the passage for the water, and re-induced tenesmic action. Injections and anodynes were suggested, to be used as the case might require —the former to keep the feces soft and facilitate the discharge.

Nov. 24th.—Patient sleeps well; has occasionally a paroxysm, lasting ten to fifteen minutes; gave ten drops tinct. opii twice to-day; pulse 86; appetite good.

Nov. 25th.—Had three returns of pain during the last twenty-four hours. I suppose these pains to be caused by the vesical irritation produced by the stone before the operation. This is the only annoying symptom in the case.

Nov. 26th.—Doing well in all respects; passed urine three times to-day naturally, two or three ounces at a time; thick, yellow pus discharging from the wound.

Nov. 27th.—Passed urine four times per urethra; bowels move freely, without pain; is taking four drops of muriatic acid three times daily, well diluted.

Dec. 2d.—The boy has improved remarkably during the fourteen days since the operation, having gained from five to ten pounds in weight. He has passed urine naturally since the twelfth day after the operation; bowels free; appetite good, and everything all right. I do not visit him any more.

Dec. 8th. The boy is reported to be up and dressed, and going about.

The stone was oval in shape, four inches in its largest circumference, and weighed 174 grains. It was of phosphatic composition.

I regard the mode of operating by dilation, and as little use of the knife as possible, an important part in the recovery of the case, and as lessening the danger of the operation materially. I believe it far better to make a dozen light cuts with a knife well guarded than to make two or three bold thrusts; for, however skillfully they may be made, the wound may be deeper than actually necessary. Even the sixteenth of an inch might turn the scale against the success of the operation, or rather of the recovery of the patient.

Obstinate Lachrymation.—We find in *Schmidt's Jahrb.* for October that a late number of the *Mon. Bl. f. Augenheilk.* contains the details of a case in which there was such profuse lachrymation as to necessitate the constant application of a cloth or handkerchief. All medication proving futile, Dr. Talko extirpated the lachrymal gland, with total relief of the distressing symptoms; but the eye continued more moist after the operation than the other eye.

Progressive Muscular Atrophy.—By J. Lockhart Clarke, M.D., F.R.S., communicated by Sir William Gull, Bart., M.D., to the Royal Medical and Surgical Society, London.

A man at the age of thirty became affected with frequent vertigo, which lasted for three years. From this period he found that he was obliged to be very slow in all his movements, and that if he attempted to move his limbs quickly they trembled very much. Subsequently he felt weakness in the left leg, so that he would suddenly fall down, without feeling giddy, and the calf of that leg began to waste. Then the muscles of the right leg wasted, and four months later he noticed wasting of the muscles of the left shoulder. He complained of severe dragging pain in the arms and legs. These symptoms gradually increased through a number of years, so that at the age of fifty-eight he was quite unable to stand, or even turn in bed, or feed himself. There was great muscular rigidity. Almost all the muscles of his body were much wasted, especially those of the upper extremities. The respiratory movements were very feeble. There was no trouble with his bladder, nor any noticeable alteration of cutaneous sensibility, but electric sensibility was almost abolished. His speech was indistinct and nasal. Deglutition became difficult, and at last almost impossible, and saliva ran from his mouth. The fibrillar tremors and rigidity of the muscles wholly disappeared during the last week of his life.

Parts of the cerebral convolutions were thickly interspersed with corpora amylacea, and many of the blood-cells of the white substance were enlarged. The cells of the grey substance were not altogether healthy. The pons Varolii was somewhat below the average size; its blood-vessels were much dilated, and in some instances had partially or wholly disappeared at particular spots, leaving empty tubular spaces. The corpora amylacea were thickly interspersed. The medulla oblongata was about one-fifth below the average size in the adult. All its nuclei were decidedly smaller than usual, and their cells were more or less affected by pigmentary degeneration. The diameter of the spinal cord was at least one-fourth less than the average in the adult. The grey substance, from one end to the other, was severely damaged by a variety of lesions and degenerations. In all regions of the cord the nerve-cells of the anterior grey substance had undergone considerable degeneration. Some of them had wholly disappeared by gradual pigmentary wasting, or by falling into heaps of granules. Of those that remained the processes were either lost or reduced considerably in size.—*The Doctor.*

